# Expression and Genetic Loss of Function Analysis of the HAT/DESC Cluster Proteases TMPRSS11A and HAT

**DOI:** 10.1371/journal.pone.0023261

**Published:** 2011-08-10

**Authors:** Katiuchia Uzzun Sales, John P. Hobson, Rebecca Wagenaar-Miller, Roman Szabo, Amber L. Rasmussen, Alexandra Bey, Maham F. Shah, Alfredo A. Molinolo, Thomas H. Bugge

**Affiliations:** 1 Oral and Pharyngeal Cancer Branch, National Institute of Dental and Craniofacial Research, National Institutes of Health, Bethesda, Maryland, United States of America; 2 Division of Extramural Activities, National Institute of Dental and Craniofacial Research, National Institutes of Health, Bethesda, Maryland, United States of America; 3 Duke University School of Medicine, Durham, North Carolina, United States of America; Stanford University, United States of America

## Abstract

Genome mining at the turn of the millennium uncovered a new family of type II transmembrane serine proteases (TTSPs) that comprises 17 members in humans and 19 in mice. TTSPs phylogenetically belong to one of four subfamilies: matriptase, hepsin/TMPRSS, corin and HAT/DESC. Whereas a wealth of information now has been gathered as to the physiological functions of members of the hepsin/TMPRSS, matriptase, and corin subfamilies of TTSPs, comparatively little is known about the functions of the HAT/DESC subfamily of proteases. Here we perform a combined expression and functional analysis of this TTSP subfamily. We show that the five human and seven murine HAT/DESC proteases are coordinately expressed, suggesting a level of functional redundancy. We also perform a comprehensive phenotypic analysis of mice deficient in two of the most widely expressed HAT/DESC proteases, TMPRSS11A and HAT, and show that the two proteases are dispensable for development, health, and long-term survival in the absence of external challenges or additional genetic deficits. Our comprehensive expression analysis and generation of TMPRSS11A- and HAT-deficient mutant mouse strains provide a valuable resource for the scientific community for further exploration of the HAT/DESC subfamily proteases in physiological and pathological processes.

## Introduction

Among the more surprising discoveries emanating from systematic genome-mining at the turn of the millennium was the unveiling of a large new family of trypsin-like membrane-anchored serine proteases, subsequently named type II transmembrane serine proteases (TTSPs) [1]. All members of this protease family feature a hydrophobic signal anchor that is located close to the amino-terminus and functions as a transmembrane domain, and a carboxy-terminal extracellular serine protease domain of the chymotrypsin (S1) fold. The signal anchor and the serine protease domain are separated by a so-called “stem region” that varies between individual TTSPs and contains an assortment of up to eleven protein domains of six different types [2], [3].

The TTSPs can be divided into four different subfamilies based on phylogenetic analysis of their serine protease domains, and this classification is supported by the composition of their stem regions and by the chromosomal localization of individual TTSP genes. These are the matriptase subfamily, the hepsin/transmembrane protease serine (hepsin/TMPRSS) subfamily, the corin subfamily, and the human airway trypsin-like protease/differentially expressed in squamous cell carcinoma (HAT/DESC) subfamily [3], [4], [5]. The human HAT/DESC subfamily comprises DESC1 (encoded by *TMPRSS11E*), HAT (encoded by *TMPRSS11D*), HAT-like 4 (encoded by *TMPRSS11F*), HAT-like 5 (encoded by *TMPRSS11B*), and TMPRSS11A (encoded by *TMPRSS11A*), [4], [6], [7], [8], [9], [10], [11], [12]. Orthologs of all five human HAT/DESC proteases are found in rodents, but rodents have two additional subfamily members (HAT-like 2 and HAT-like 3, encoded by, respectively, the *Desc4* and *Tmprss11c* genes) that are not found in humans or chimpanzees. This divergence of the primate and rodent HAT/DESC protease complement appears to be caused by gene loss in primates, rather than expansion of the rodent *DESC* cluster, as pseudogene orthologs of the rodent *Desc4* and *Tmprss11c* genes are present in the human and chimpanzee genomes [8], [10].

All members of the HAT/DESC subfamily possess a structurally identical stem region that is composed of a single sea urchin sperm protein, enteropeptidase, agrin (SEA) domain and they display high overall amino acid sequence identity in all their domains, suggesting a potential for partial functional redundancies [7]. Systematic side-by-side comparisons of their expression to support this suggestion, however, have not been performed.

At the time of the discovery of the TTSPs, a physiological function was only established for a single member of the family; the digestive protease enteropeptidase [13]. Within the last decade, however, gene targeting studies in mice and gene mapping of humans with autosomal recessive inherited diseases have provided dramatic progress towards assigning physiological functions for individual members of the matriptase, TMPRSS, and corin subfamilies [14], [15], [16], [17], [18], [19], [20], [21], [22], [23], [24], [25], [26]. Comparatively less, however, is known about the physiological functions of members of the HAT/DESC subfamily. HAT was originally purified from the sputum of patients with chronic airway disease [27]. It has been proposed to execute a diverse array of functions in epithelial tissues through the cleavage of specific substrates. These proposed functions include fibrinogenolysis leading to suppression of coagulation, [28], proteolytic activation of protease activated receptor (PAR)-2, [29], [30], [31], [32], and urokinase plasminogen activator cleavage with modulation of cell adhesion and migration [33]. Moreover, a secreted variant of HAT was reported to be the processing enzyme for pro-γ-melanotropin in the rat adrenal gland [34], [35].

In this study, we have performed a combined expression and genetic analysis of the HAT/DESC subfamily proteases. We show that members of the family are coordinately expressed in mice and humans, and that the ablation of the *Tmprss11a* gene, encoding TMPRSS11A and of the *Tmprss11d* gene, encoding HAT, does not adversely affect embryonic development, health, and long-term survival in the absence of external challenges or additional genetic deficits. The study suggests that functional redundancies exist between HAT/DESC proteases in maintaining basic homeostatic functions and it provides two valuable new mutant mouse strains for further functional dissection of this large relatively unexplored protease subfamily.

## Materials and Methods

### Ethics Statement

All animal work was performed in accordance with protocols approved by the National Institute of Dental and Craniofacial Research Animal Care and Use Committee (Animal Study Proposal Number: 08-465).

### HAT/DESC TTSP subfamily gene expression analysis in mouse and human organs

Mouse total RNA was prepared from tissues of six-month-old wild-type mice by extraction in Trizol reagent (Gibco-BRL, Carlsbad, CA), as recommended by the manufacturer. The “First Choice Human Total RNA Survey Panel” (Ambion-Applied Biosystems, Austin, TX) and human salivary gland total RNA (Clontech-BD Biociences, Palo Alto, CA) were used to analyze gene expression in humans. First strand cDNA synthesis was performed from 1 µg of total RNA using a RetroScript kit *(Ambion, Inc. Austin TX)* and an oligo dT primer according to the manufacturer's instructions. The subsequent PCR was performed with a “Taq PCR Master Mix” kit (Qiagen, Valencia, CA) using gene-specific primers designed to anneal to separate exons of each of the mouse or human HAT/DESC genes (see Table 1 and Table 2 for primer sequences). All PCRs were run for 35 cycles of 1 min denaturation at 94°C, 1 min annealing at 57°C for mouse genes and 55°C for human genes, and 1 min elongation at 72°C. Amplicons were analyzed by agarose gel electrophoresis.

**Table 1:** Sequences of primers used for expression analysis of HAT/DESC genes in mouse tissues.

*Tmprss11a*	
Forward	5′-TCTAGTGCAGTTTTCTCCC-3′
Reverse	5′-CTTTTGACCACAGTTGTCTC-3′
*Tmprss11b*	
Forward	5′-GAACATCATGATGACGTTGC-3′
Reverse	5′-TGACTCTGCCACATTCATGC-3′
*Tmprss11c*	
Forward	5′-CACGAGAACTACAGTTACCC-3′
Reverse	5′-CATTCCAGGTGTGATCATGC -3′
*Tmprss11d*	
Forward	5′-CTGTCGCATATGTTACAGG-3′
Reverse	5′-ACAATGCCCACAACAAACC-3′
*Tmprss11e*	
Forward	5′-CAACCTCGAAAACTGACG-3′
Reverse	5′-ACATCCTAGGAGTGATGGC-3′
*Tmprss11f*	
Forward	5′-GTGGTTCAGAGAGTCTGCC-3′
Reverse	5′-GTCACTCTTGTGTAGACTCC-3′
*Desc4*	
Forward	5′-CGACTTTTCAAGTCTTGCC-3′
Reverse	5′-CGATAATGAGTCACTCTGG-3′
*Rps15*	
Forward	5′-TTCCGCAAGTTCACCTACC-3′
Reverse	5′-CGGGCCGGCCATGCTTTACG-3′

**Table 2:** Sequences of primers used for expression analysis of HAT/DESC genes in human tissues.

*TMPRSS11A*	
Forward	5′-CAAGAGAGTACGACATTGC-3′
Reverse	5′-CACGTATCTTTCAGATCCC-3′
*TMPRSS11B*	
Forward	5′-GTACATCGAGTTTGTCTTCC-3′
Reverse	5′-TAGGATGAACTAGTGGTCC-3′
*TMPRSS11D*	
Forward	5′-TCACTCGAGTATACACTCC-3′
Reverse	5′-TCACCTTTACCAAAGATATCC-3′
*TMPRSS11E*	
Forward	5′-CTCACTATTCCAGCAAGG-3′
Reverse	5′-ATGGACTGCTTCCTTTGG-3′
*TMPRSS11F*	
Forward	5′-ACCTAAAACAAGTGTGTTCG-3′
Reverse	5′-TCGATACTTAGTTACTCTGG-3′
*RPS15*	
Forward	5′-TTCCGCAAGTTCACCTACC-3′
Reverse	5′-CGGGCCGGCCATGCTTTACG-3′

### Gene targeting

#### Tmprss11a

Mice carrying a mutant *Tmprss11a* allele (*Tmprss11a^tm1Dgen^*) were generated by Deltagen Inc. (San Mateo, CA) and acquired from the Jackson Laboratories through the “NIH initiative supporting placement of Deltagen, Inc., mice into public repositories”. Gene targeting was performed by homologous recombination in 129S1/SvImJ x129X1/SvJ-derived R1 embryonic stem cells [36] using a targeting vector that replaces nucleotides 780 to 909 of the *Tmprss11a* mRNA with a neomycin transferase expression cassette. The cassette was flanked by homologous sequences of 1 kb (5′ arm) and 3 kb (3′ arm). Correct targeting was verified by Southern blot hybridization of Eco RI digested DNA located external to the targeting vector. Chimeric mice were bred to C57BL/6J mice and scored for germ line transmission. Genotyping of mice was performed by PCR using the primers 5′-CCCACCGCCATAGTAAAGTGCTCCG-3′ (nucleotides 48920–48944, NC_000071.5) in combination with either 5′-GCAATTCAAACCCTCGCCAATGGAC-3′ (nucleotides 48729–48753) for detection of the endogenous allele or a neomycin-specific primer 5′-GGGTGGGATTAGATAAATGCCTGCTCT-3′ for detection of the targeted allele.

#### Tmprss11d

The chromosomal insertion and chromosome engineering resource (MICER) insertional gene targeting vector MHPN265D14 containing nucleotides 86759113–86767511 of chromosome 5 from 129S5/SvEv^Brd^ mice was obtained from the Welcome Trust Sanger Institute, Cambridge, UK [37]. The targeting vector was linearized with NdeI (nucleotide 86759442) and introduced into R1 embryonic stem cells by electroporation using 0.4 kVolts/25 uFD with a time constant of 0.4 msec. The embryonic stem cell clones were grown in the presence of 350 µg/ml G418 for eight days. One hundred and fifty five G418-resistant embryonic stem cell clones were expanded and screened for targeted insertion of the vector into the *Tmprss11d* locus by Southern blot hybridization of SpeI-digested genomic DNA using a ^32^P-labeled 482 bp probe spanning nucleotides 86768086 to 867676604 of chromosome 5, external to the targeting vector sequences. A correctly targeted embryonic stem cell clone was injected into the blastocoel cavity of C57BL/6J-derived blastocysts and implanted into pseudopregnant females. Chimeric male offspring were bred to NIH Black Swiss females (Taconic Farms, Germantown, NY) to generate heterozygous offspring. These mice were subsequently interbred to generate *Tmprss11d* ^−/−^ and littermate progeny for analysis. Genotyping of mice was performed by Southern blot with a probe external to the targeting vector sequences (86757395–86757810 of chromosome 5) that were amplified by PCR using the primers 5′-AGGACTATTGGGAGTGCC-3′ and 5′-GAAAATCGGAAGAGTGCC -3′.

### Analysis of transcripts from mutant Tmprss11a and Tmprss11d alleles

Total RNA was prepared from tongues of 701 and 746 days-old *Tmprss11a^−/−^* and *Tmprss11a^+/+^* mice, respectively, and from tracheas of 194 days-old *Tmprss11d^−/−^ and Tmprss11d^+/+^* mice. After euthanization, tongues and tracheas were snap-frozen in liquid nitrogen, ground to a fine powder with a mortar and pestle, and RNA was extracted in Trizol reagent (Gibco-BRL) as recommended by the manufacturer. The RNA was reverse transcribed and amplified by PCR using the RETROscript™ Kit as recommended by the manufacturers. First strand cDNA synthesis was performed using an Oligo DT primer. PCR amplification of *Tmprss11a* transcripts was performed using primers that amplify nucleotides 762 to 939 of the *Tmprss11a* mRNA (NM_001033233.2), which includes the deleted portion of the sequence (nucleotides 780 to 909), using the forward primer DESC-3delF (5′-GACTCTTAGTTTTGGAACAAC-3′) and the reverse primer DESC-3delR (5′-TTCATCCGAAAAGGTGACTC-3′). The primers E2–E9 forward: (5′-GGATATGGCACCCACAACAGAG-3′) and E2–E9 reverse: (5′-ACCCGTTTTCGAAGCAATCCA-3′) were used to amplify transcripts containing exons 2–9 (nucleotides 5–388), E2–E9 forward and E2–E5 reverse (5′-AACCAGTGAGACATCAGCTGG-3′) to amplify transcripts containing exons 2–5 (nucleotides 5–141), and E8–E9 forward (5′-GGGAATCCCAAAATGAGCTC-3′) and E2–E9 reverse to amplify transcripts containing exons 8 and 9 (nucleotides 289–388). Tmprss11d transcripts were amplified using a forward primer that anneals to nuclotides 327–352 (NM_145561.2) and a reverse primer that anneals to nucleotides 285–308 (5′-ATCAGGACACATGTTGTCAAACTAAG-3′ and 5′-TGATCCTCGAAATTCATCAGTAAT-3′, respectively).

### Analysis of postnatal growth and long-term health

Prospective cohorts of mice were housed in standard HEPA-filtered mixed genotype cages containing up to five mice. The mice received standard mouse chow and water *ad libitum* and were observed twice daily for moribundity or death. Mice were scored as diseased the morning of being found dead or after being euthanized due to moribundity. Weight gain and outward appearance were systematically investigated and recorded every two weeks. Mice were euthanized at the end of the observation period, and gross autopsy was performed by a pathologist (A. M.) unaware of animal genotype. Organs then were dissected, fixed for 24 h in 4% paraformaldehyde in water, processed into paraffin, sectioned into parallel sagittal sections, and stained with H&E. The sections were analyzed under light microscopy and analyzed by K. U. S. and A. M.

## Results

### Expression of HAT/DESC cluster transcripts in mice and humans

**Figure 1:**
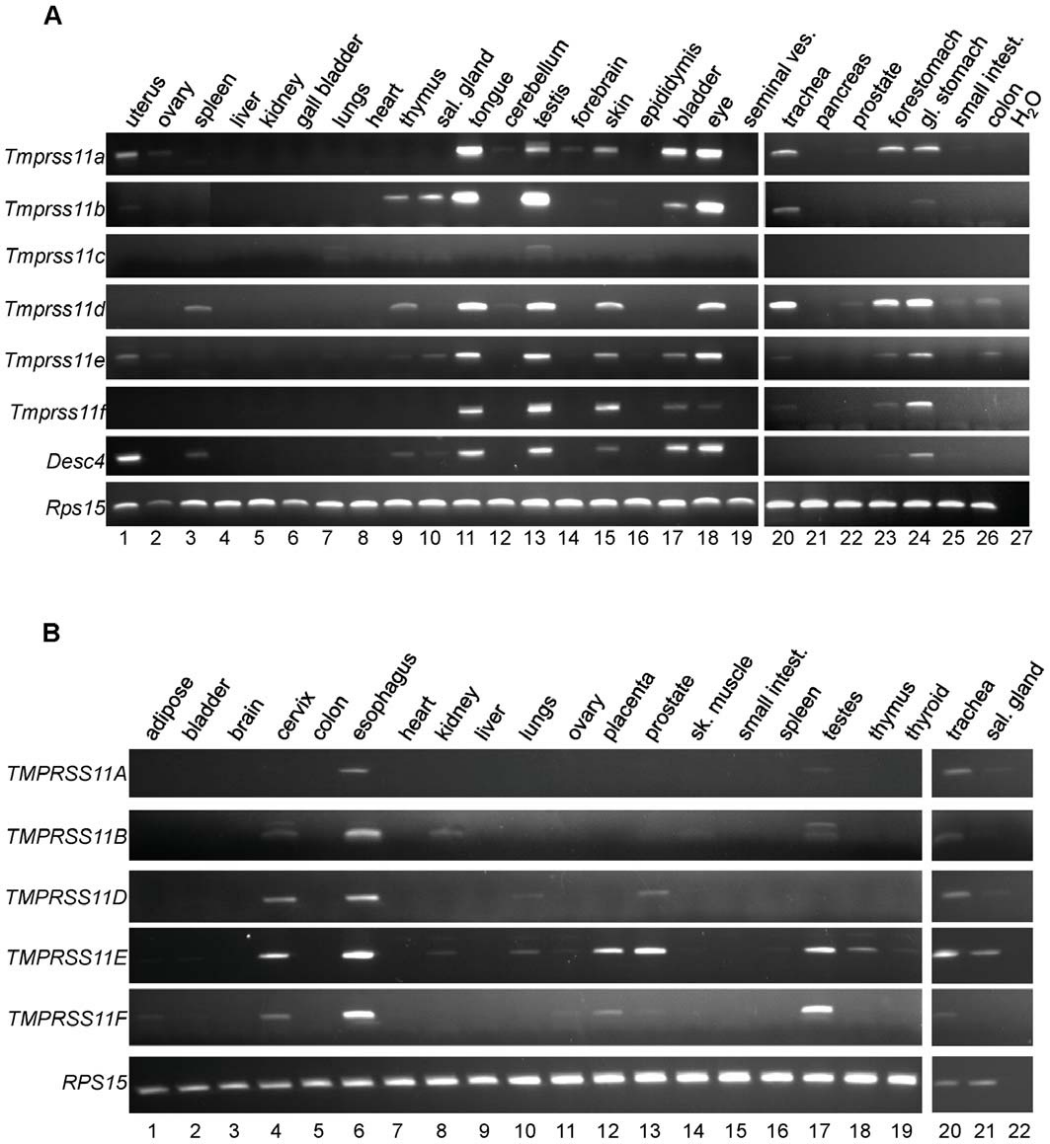
Distribution of HAT/DESC subfamily gene transcripts in mouse and human organs. PCR analysis of expression of mouse *Tmprss11a-f* and *Desc4*, and expression of human *TMPRSS11A*, *B* and *D*–*F* using total RNA reverse transcribed from either 26 mouse organs of young adult mice (A) or from 21 human organs (B), as indicated. Mouse (*Rps15*) and human *(RPS15)* ribosomal S15 protein genes were used as controls. All amplicons displayed their predicted molecular weights.

Limited information as to the expression of the HAT/DESC subfamily proteases could be obtained from searching the “Eurexpress Transcriptome Atlas Database for Mouse Embryo” [38]. Only *Tmprss11d* displayed detectable expression in epithelia of the oral cavity, esophagus, and the anterior and posterior parts of the naris, whereas *Tmprss11e* and *Tmprss11f* displayed no signal, and no entries were available for *Tmprss11a*, *Tmprss11b*, *Tmprss11c*, and *Desc4*. We, therefore, performed a comprehensive side-by-side comparison of the expression of each of the seven mouse HAT/DESC subfamily genes and each of the five human HAT/DESC subfamily genes in a wide range of adult organs by RT-PCR analysis (Figure 1A and B). *Tmprss11c* was only faintly expressed in lungs and testis (Figure 1A, lanes 7 and 13) and was not detected in the other mouse 24 organs analyzed. The remaining six mouse subfamily genes displayed a coordinated pattern of expression. For example, little or no transcripts of each of the six genes could be detected in gall bladder, heart, kidney, liver, lungs, ovary, pancreas, and seminal vesicle (Figure 1A, lanes 2, 4–7, 8, 16, 19, 21). Conversely, transcripts of all six genes were present in eye, testis, glandular stomach, and the tongue (Figure 1A, lanes 11, 13, 18, and 24), and transcripts of five of these six genes were present in bladder, forestomach, skin, and trachea (Figure 1A, lanes 15, 17, 20, 23). Only five mouse organs displayed transcripts for a single HAT/DESC protease (cerebellum, epididymis, forebrain, prostate, and small intestine (Figure 1A, lanes 12, 14, 16, 22, 25).

A similar overlapping pattern of expression was observed for the five human HAT/DESC subfamily genes. No transcripts for either of the five human HAT/DESC protease-encoding genes could be detected in brain, colon, heart, and liver (Figure 1B, lanes 3, 5, 7, and 9), whereas transcripts of all five genes were present in esophagus and trachea (Figure 1B, lanes 6 and 20), four the five genes were present in cervix and testis (Figure 1B, lanes 4 and 17), three of the five genes were present in prostate and salivary gland (Figure 1B, lanes 13 and 17), and two of the five genes were present in kidney, lungs, ovary, and placenta (Figure 1B, lanes 8, 10, 11, and 12), and transcripts of only one gene was present in spleen and thymus (Figure 1B, lanes 16, 18). A variable degree of species conservation in expression of mouse and human HAT/DESC transcripts was evident when comparing the fifteen organs that were analyzed in both mouse and human. Most consistent was the expression of five of seven mouse subfamily members and five of five human subfamily members in the trachea, and the low or absent expression both mouse and human genes in brain, heart, and liver.

### Generation of TMPRSS11A and HAT-deficient mice

**Figure 2:**
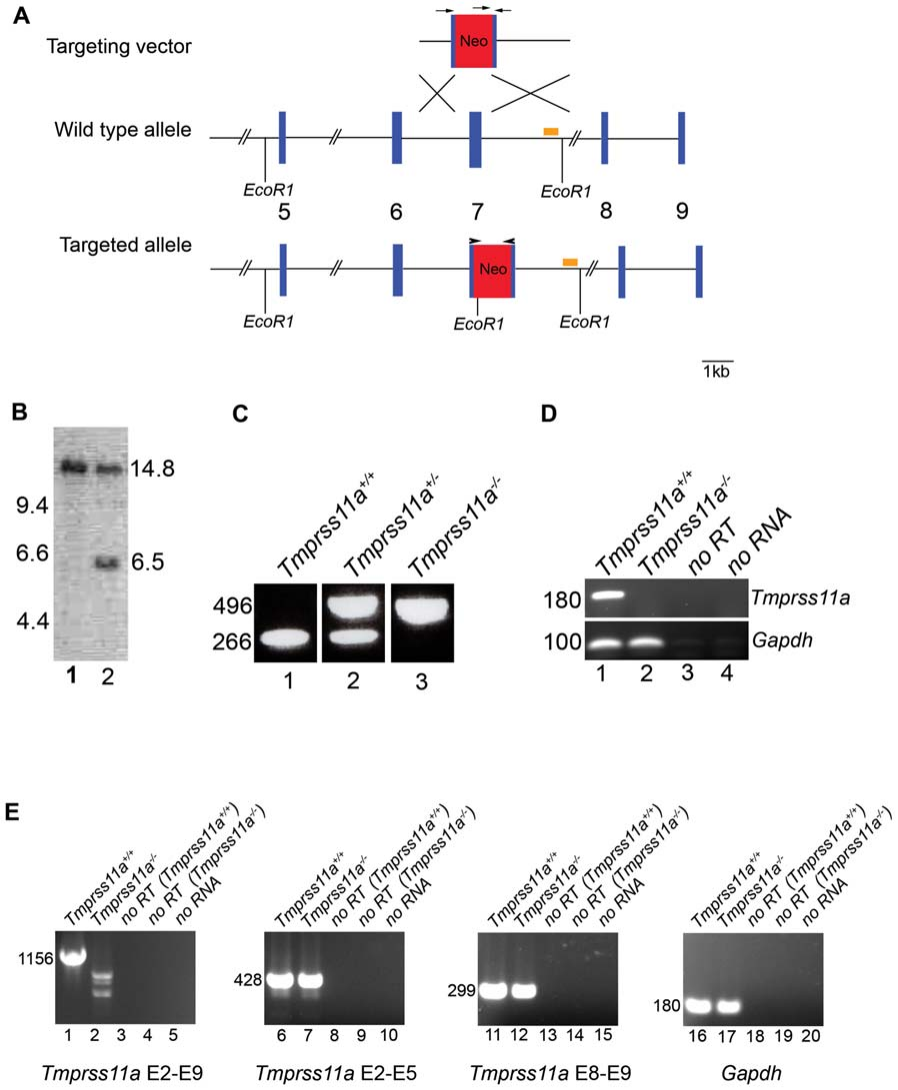
Generation of TMPRSS11A-deficient mice. A. Schematic structure of the gene targeting replacement vector (top), wildtype *Tmprss11a* gene (middle), and targeted *Tmprss11a* gene (bottom). Position of Eco RI restriction enzyme cleavage sites used for digestion of genomic DNA for Southern blot analysis, position of Southern blot probe (bar), and positions of primers used for analysis of wildtype and mutant *Tmprss11a* alleles (arrows) and transcripts (arrowheads) are indicated. B. Southern blot hybridization of Eco-RI digested DNA from a control (lane 1) and a targeted (lane 2) embryonic stem cell clone. The positions of wildtype (14.8 kb) and mutant *Tmprss11a* (6.5 kb) alleles are indicated on the right. Positions of molecular weight markers (kb) are indicated at left. C. PCR analysis of tail biopsy DNA of offspring from interbred *Tmprss11a^+/−^* mice. The position of wildtype (496 bp) and mutant *Tmprss11a* (266 bp) alleles are indicated. D. RT-PCR analysis of *Tmprss11a* mRNA transcripts from tongue of *Tmprss11a^+/+^* (lane 1) and *Tmprss11a^−/−^* (lane 2) mice using the exon 7-flanking primer pair indicated with arrowheads in A. Lanes 3 and 4, no reverse transcriptase and no RNA added to the reactions, respectively. Bottom panel. Amplification of *Gapdh* mRNA demonstrating the integrity of the cDNA preparation. E. RT-PCR amplication of *Tmprss11a* mRNA transcripts from tongue of *Tmprss11a^+/+^* (lanes 1, 6, 11, and 16) and *Tmprss11a^−/−^* (lane 2, 7, 12, and 17) mice using primer pairs capable of amplifying exons 2–9 (lanes 1–5), 2–5 (lanes 6–10), 8–9 (lanes 11–15), and *Gapdh* (lanes 16–20). Reverse transcriptase was omitted from the reactions in lanes 3, 4, 8, 9, 13, 14, 18, and 19. No RNA was added to reactions in lanes 5, 10, 15 and 20. Transcript size is indicated left.

Of the seven mouse HAT/DESC subfamily genes analyzed above, *Tmprss11a*, encoding TMPRSS11A (also known as DESC3 and HAT-like 1) and *Tmprss11d*, encoding human airway trypsin-like serine protease (HAT) (also known as adrenal serine protease) were among the genes whose transcripts could be found in the largest number of organs. To further explore the function of the two membrane anchored serine proteases in development and postnatal tissue homeostasis, we next determined the phenotypic consequences of ablation of either TMPRSS11A or HAT in mice. Care was taken to ensure that the selected targeting strategies resulted in the generation of null alleles, as no in-house generated or commercially available antibodies proved capable of detecting TMPRSS11A or HAT in mouse tissues (data not shown). The *Tmprss11a* gene was disrupted by replacing 129 nucleotides of exon seven with a neomycin transferase gene expression cassette using homologous recombination in embryonic stem cells (Figure 2A). The deleted exon seven sequence encodes amino acids 216–258 of TMPRSS11A, which includes Asp243 that forms part of the catalytic triad of the serine protease. Southern blot of targeted embryonic stem cells (Figure 2B), as well as PCR of genomic DNA (Figure 2C) and RT-PCR analysis (Figure 2D) of tongues of mice bred to homozygosity for the mutant allele confirmed the absence of both *Tmprss11a* gene sequences and mRNA transcripts containing exon seven. RT-PCR analysis using primer pairs capable of spanning exons 2–9, 2–5, and 8–9 (Figure 2E) demonstrated that the targeted generated transcripts with a capacity to produce a catalytically inactive truncated protein.

**Figure 3:**
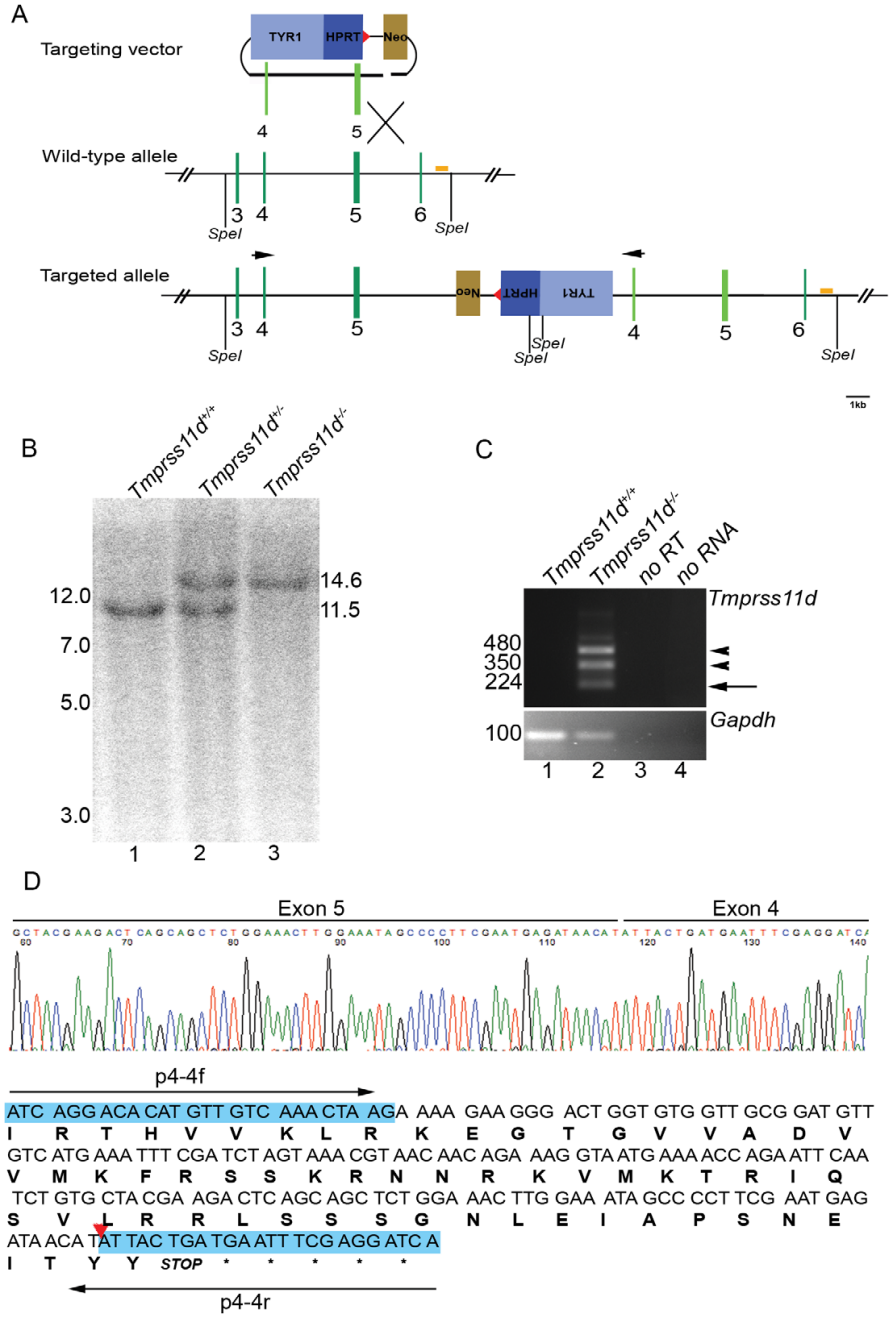
Generation of HAT-deficient mice. A. Schematic structure of the targeted insertion vector (top), wildtype *Tmprss11d* gene (middle), and targeted *Tmprss11d* gene (bottom). Position of Spe I restriction enzyme cleavage sites used for digestion of genomic DNA for Southern blot analysis, position of Southern blot probe (bar), and primers used for analysis of mutant *Tmprss11d* transcripts (arrows) are indicated. Red triangle indicates loxP site. B. Southern blot hybridization of Spe I-digested tail biopsy DNA from offspring from interbred *Tmprss11d^+/−^* mice. The position of wildtype (11.5 kb) and mutant *Tmprss11d* (14.6 kb) alleles are indicated on the right. Positions of molecular weight markers (kb) are indicated at left. C. Analysis of mRNA transcripts generated by the mutant *Tmprss11d* allele. Top panel. RT-PCR analysis of *Tmprss11d* mRNA transcripts from tracheas of *Tmprss11d^+/+^* (lane 1) and *Tmprss11d^−/−^* (lane 2) mice using the exon 4 primer pair shown in A. Lanes 3 and 4, no reverse transcriptase and no RNA added to the reaction, respectively. Positions of amplicons revealed by subsequent sequencing to be derived from exon 4-exon 5-exon 4 spliced mutant mRNA (arrow), and amplicons revealed by subsequent sequencing to represent transcripts to derive from the targeting cassette (arrowheads) are shown. Bottom panel. Amplification of *Gapdh* mRNA demonstrating the integrity of the cDNA preparation. Positions of molecular weight markers (bp) are indicated at left. D. Sequence analysis of the exon 4-exon 5-exon 4 amplicon. Exon 4-derived sequences are shaded blue. The shift of the reading frame of the mutant mRNA (red triangle) and the associated introduction of a stop codon is indicated. Positions of primers used for PCR amplication are indicated with arrows.

The *Tmprss11d* gene was disrupted by introducing a duplication of exons four and five and inserting a tyrosinase-neomycin expression cassette between the duplicated exons using a Mutagenic Insertion and Chromosome Engineering Resource (MICER) targeted insertion vector (Figure 3A). This duplication introduces a frameshift mutation in the SEA domain located upstream of the serine protease domain of HAT. RT-PCR using a primer pair complementary to exon four confirmed the presence of both the duplicated mutant transcripts in addition to cryptic transcripts originating from the tyrosinase cassette (Figure 3C and D). To further ensure that the employed targeting strategy resulted in a null allele, we next performed RT-PCR with primer pairs that would be capable of detecting any alternatively-spliced *Tmprss11d* transcripts with the hypothetic potential to encode a functional protease (defined as transcripts that would encode the signal anchor, propeptide, and catalytic triad). No alternative transcripts were detected by this analysis (data not shown).

### Effects of TMPRSS11A and HAT ablation on development, health, and long-term survival

**Figure 4:**
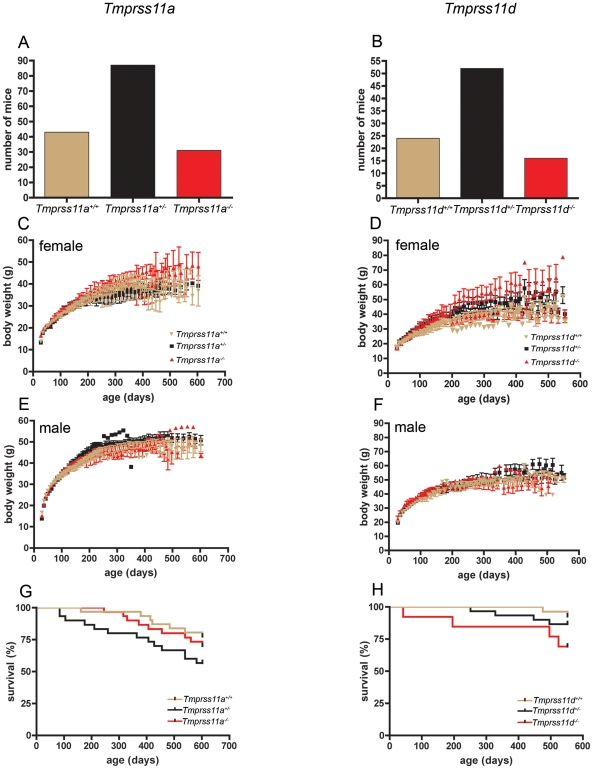
Development, growth, and survival of TMPRSS11A- and HAT-deficient mice. A and B. Genotype distribution of weaning-age offspring of interbred *Tmprss11a^+/−^* (A) and *Tmprss11d^+/−^* (B) mice. C–F. Post-weaning weight gain of cohorts of littermate *Tmprss11a^+/+^* (golden triangles, N = 16), *Tmprss11a^+/−^* (black squares, N = 15), and *Tmprss11a^−/−^* (red triangles, N = 15) females (C), *Tmprss11a^+/+^* (golden triangles, N = 15), *Tmprss11a^+/−^* (black squares, N = 15), and *Tmprss11a^−/−^* (red triangles, N = 15) males (E), *Tmprss11d^+/+^* (golden triangles, N = 12), *Tmprss11d^+/−^* (black squares, N = 15), and *Tmprss11d^−/−^* (red triangles, N = 6) females (D), *Tmprss11d^+/+^* (golden triangles, N = 15), *Tmprss11d^+/−^* (black squares, N = 15), and *Tmprss11d^−/−^* (red triangles, N = 7) males (F). G and H. Survival of prospective cohorts of littermate *Tmprss11a^+/+^* (golden lines, N = 30), *Tmprss11a^+/−^* (black lines, N = 30), and *Tmprss11a^−/−^* (red lines, N = 30) mice (G) and *Tmprss11d^+/+^* (golden lines, N = 27), *Tmprss11a^+/−^* (black lines, N = 30), and *Tmprss11a^−/−^* (red lines, N = 13) (H) mice that were followed for at least 500 days.

**Figure 5:**
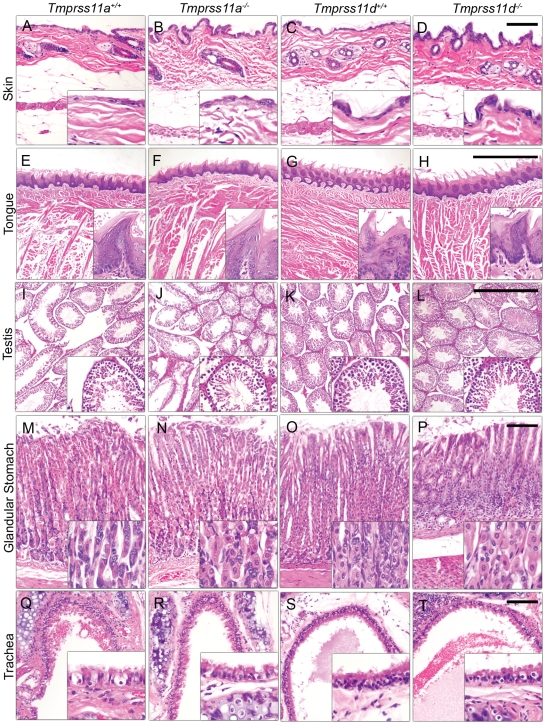
Microscopic appearance of tissues from aging HAT- and TMPRSS11A-deficient mice. Representative examples of the histological appearance of hematoxylin and eosin-stained sections of the skin (A–D), tongue (E–H), testis (I–L), glandular stomach (M–P), and trachea (Q–T) from 701–747 days-old *Tmprss11a^+/+^* (A, E, I, M, and Q) and littermate *Tmprss11a^−/−^* (B, F, J, N, and R) mice, and from 671–678 days-old *Tmprss11d^+/+^* (C, G, K, O, and S) and littermate *Tmprss11d^−/−^* (D, H, L, P, and T) mice. Inserts show higher magnification of boxed areas. Size bars in inserts are 100 µm.

Genotype analysis of 161 offspring from crosses of mice heterozygous for the mutant *Tmprss11a* allele, and of 92 offspring from crosses of mice heterozygous for the mutant *Tmprss11d* allele showed that HAT and TMPRSS11A were both dispensable for development (Figure 4A and B). Thus, the distribution of wildtype offspring (*Tmprss11a^+/+^*, *Tmprss11d^+/+^*), offspring heterozygous for the targeted alleles (*Tmprss11a^+/−^*, *Tmprss11d^+/−^*), and offspring homozygous for the targeted allele (*Tmprss11a^−/−^*, *Tmprss11d^−/−^*) did not deviate significantly from the expected 1∶2∶1 Mendelian distribution, although slightly fewer *Tmprss11a^−/−^* and *Tmprss11d^−/−^* offspring were detected (P>0.05, Chi-square test, two-tailed). *Tmprss11a^−/−^* and *Tmprss11d^−/−^* mice both appeared outwardly normal at birth and at weaning (data not shown). To determine the effect of loss of TMPRSS11A and HAT on overall health and survival, we next established prospective cohorts of *Tmprss11a^−/−^* mice (15 females and 15 males) and their *Tmprss11a^+/−^* (15 females and 15 males) and *Tmprss11a^+/+^* (16 females and 15 males) littermates, as well as of *Tmprss11d^−/−^* mice (six females and seven males) and their *Tmprss11d^+/−^* (15 females and 15 males) and *Tmprss11d^+/+^* (12 females and 15 males) littermates. The weight and outward appearance of each mouse enrolled in the cohorts was recorded bi-weekly for at least 455 days, until death, or until moribundity of the mouse necessitated euthanization to comply with animal study protocol endpoints. Neither TMPRSS11A or HAT deficiency significantly affected weaning weights or post-weaning weight gain of either females or males. Furthermore, both protease-deficient mutant mouse strains displayed similar long-term survival (Figure 4G and H). Full necropsies and microscopic examination if all tissues of five female mice and five male mice enrolled in the two cohorts were performed after their euthanization (Tables 3 and 4 and Figure 5). A number of mostly age-related pathologies, including leukemia/lymphoma, carcinoma, tissue atrophy/necrosis, hyperplasia, thrombosis, hemorrhage, and chronic inflammation were prevalent, but these generally did not correlate with genotype. However, prostate hyperplasia was observed in three *Tmprss11a^−/−^* mice, but not in *Tmprss11a^+/+^* littermates. Likewise all *Tmprss11d^−/−^* females presented with lymphoma, whereas this was observed only in three *Tmprss11d^+/+^* females. Taken together, our study shows that TMPRSS11A and HAT are dispensable for mouse development to term, postnatal growth, long-term health, and survival in the absence external challenges and other genetic deficits.

**Table 3:** Pathological findings in tissues from aging TMPRSS11A-deficient mice.

Group	No. mice	Age (Days) Median; Range	Gender	Pathology	Frequency
*Tmprss11a^+/+^*	5	764; 746–769	Male	Atrophic skin	3 (60%)
				Lymphoma	2 (40%)
				Focal dysplastic changes in the stomach	1 (20%)
				Small adenoma of lung	1 (20%)
				Hepatocyte vacuolization and congestion	1 (20%)
				Haemosiderin deposition in the liver	1 (20%)
				Peribronchial lymphocytic infiltration	1 (20%)
				Dilated prostate	1 (20%)
				Corpora amilacea in the prostate	1 (20%)
				Reactive chronic hepatitis	1 (20%)
				Atrophic cortical area in the kidney	1 (20%)
*Tmprss11a^−/−^*	5	745; 700–768	Male	Atrophic skin	3 (60%)
				Prostate hyperplasia	3 (60%)
				Atrophic testis	2 (40%)
				Dilated seminal glands	2 (40%)
				Chronic inflammation of the salivary glands	2 (40%)
				Preputial gland chronic inflammation	2 (40%)
				Hepatitis	1 (20%)
				Focal steatosis in the liver	1 (20%)
				Lymphoma	1 (20%)
				Chronic congestion of lung	1 (20%)
				Papillary adenoma of lung	1 (20%)
				Hyperplastic seminal glands	1 (20%)
				Liver hemorrhage	1 (20%)
				Chronic inflammation of kidney	1 (20%)
				Stomach dysplasia	1 (20%)
*Tmprss11a^+/+^*	5	732; 701–767	Female	Necrotic peritoneum	2 (40%)
				Atrial calcified thrombosis	1 (20%)
				Liver hyperplasia/extramedullary hematopoiesis	1 (20%)
				Megakaryoblastic leukemia	1 (20%)
				Cavernous hemangioma in uterus	1 (20%)
				Uterus cystic hyperplasia	1 (20%)
				Lymphadenitis granulomatosa in the lymphnode	1 (20%)
				Adenoma of lung	1 (20%)
*Tmprss11a^−/−^*	5	724; 681–768	Female	Uterus cystic hyperplasia	3 (60%)
				Atrophic skin	2 (40%)
				Hepatic degeneration	2 (40%)
				Lung adenoma	2 (40%)
				Necrotic peritoneum	2 (40%)
				Lymphoma	1 (20%)
				Lung hemorrhage	1 (20%)
				Spleen hemorrhage	1 (20%)
				Atrophic mammary gland	1 (20%)
				Vacuolization of hepatocytes	1 (20%)

**Table 4:** Pathological findings in tissues from aging HAT-deficient mice.

Group	No. mice	Age (Days) Median; Range	Gender	Pathology	Frequency
*Tmprss11d^+/+^*	5	764; 746–769	Male	Liver fat degeneration	3 (60%)
				Chronic inflammation of the preputial glands	2 (40%)
				Dilated bladder	2 (40%)
				Prostate concretions and inflammation	2 (40%)
				Salivary glands dysplasia	1 (20%)
				Kidney angiitis	1 (20%)
				Focal ossification in spleen with angiitis	1 (20%)
				Adenoma of the lung	1 (20%)
				Papillary hyperplasia of thyroid	1 (20%)
				Lymphoma	1 (20%)
				Hypertrophy of seminal glands	1 (20%)
				Testis calcification	1 (20%)
*Tmprss11d^−/−^*	3	745; 700–768	Male	Lymphoma	2 (40%)
				Stomach focal dysplastic changes	2 (40%)
				Chronic inflammation of prostate	2 (40%)
				Adenoma of the lung	1 (20%)
				Chronic reactive hepatitis	1 (20%)
				Liver steatosis	1 (20%)
*Tmprss11d^+/+^*	5	656; 633–705	Female	Liver fat degeneration	3 (60%)
				Lymphoma	3 (60%)
				Uterus cystic hyperplasia	1 (20%)
				Deciduoma (endometrial polyp)	1 (20%)
				Polyposis dysplasia of the stomach	1 (20%)
*Tmprss11d^−/−^*	5	656; 633–705	Female	Lymphoma	5 (100%)
				Liver fat degeneration	3 (60%)
				Liver necrosis	1 (20%)
				Adenoma of the lung	1 (20%)
				Stomach dysplasia	1 (20%)
				Squamous metaplasia of mammary glands	1 (20%)
				Hyperplasia of Langerhans islands	1 (20%)
				Uterus cystic hyperplasia	1 (20%)

## Discussion

The pace with which the physiological functions of the recently emerged family of TTSPs have been elucidated has been rapid. Through loss of function studies in mice, humans, and fish, a diverse array of fundamental cell and developmental functions have been established for members of the matriptase, hepsin/TMPRSS, and corin subfamilies, including tissue morphogenesis, epithelial barrier function, ion and water transport, cellular iron export, and blood pressure regulation. No similar information, however, is as yet available for members of the large HAT/DESC subfamily of TTSPs.

In this study, we performed the first comprehensive expression and loss of function genetic analysis of members of the HAT/DESC subfamily. We found that transcripts of the seven functional murine and the five functional human HAT/DESC protease-encoding genes were present in a large number of organs. In both mice and humans, members of the subfamily displayed coordinated gene expression, as revealed by the presence of transcripts of all or most HAT/DESC genes in some organs, and a corresponding absence of expression or expression of only a single gene in several other organs.

Phenotypic analysis of mice carrying null mutations in two of the most widely expressed HAT/DESC subfamily genes, *Tmprss11a* and *Tmprss11d*, did not reveal an effect of the loss of either of the genes on development, postnatal growth or long-term health, although prostate hyperplasia was seen only in *Tmprss11a^−/−^* males, and the incidence of lymphoma was lower in *Tmprss11d^+/+^* females in small cohorts of older animals subjected to detailed histopathological examination.

While the strategy used to target *Tmprss11a* and *Tmprss11d* precludes both genes from generating a functionally active protease, transcripts potentially capable of generating truncated versions of TMPRSS11A and HAT were produced from each of the mutant alleles. It is therefore formally possible that each of these truncated proteins would be capable of carrying out some non-proteolytic function, although such an auxiliary function has not been described to data for a membrane-anchored serine protease [39].

In light of the aforementioned coordinated expression of members of the subfamily and the high amino acid identity between individual HAT/DESC proteases, it is tempting to speculate that functional redundancies may exist within the family during development and in the maintenance of basic homeostasis. However, even the prostate, which displayed expression of only *Tmprss11d*, was unremarkable in *Tmprss11d*-deficient mice.

Genetic analysis aimed at delineating potential functional redundancies of HAT/DESC proteases poses particular technical problems, chiefly due to the tight clustering of their corresponding genes, which makes simple interbreeding of mice with individual gene deficiencies to generate mice with multiple gene deficiencies a practical impossibility. Rather, the sequential targeting of embryonic stem cells [40] or the use of novel zinc-finger gene targeting strategies would have to be employed [41]. The latter strategy would allow for rapid generation of mice with combined null mutations in HAT/DESC cluster genes. It should be noted, however, that the high amino acid identity of TTSP family genes and the tight clustering of their cognate genes does not necessarily imply extensive functional redundancy. Thus, of the five hepsin/TMPRSS subfamily members whose homozygous inactivation has been reported in mice or humans (*HPN*, *TMPRSS2*, *TMPRSS3*, *TMPRSS5*, and *PRSS7*) only the loss of *TMPRSS2* was not associated with a spontaneous phenotype [19], [21], [42], [43], [44], [45].

In summary, our current study constitutes a first step towards genetically deciphering the functions of the HAT/DESC subfamily of TTSPs. The comprehensive expression analysis and availability of TMPRSS11A- and HAT-deficient mice will provide a valuable resource for the scientific community for additional functional exploration of the physiological and pathological roles of this fascinating protease family.
